# Systematic review and case report: Systemic lupus erythematosus with renal tubular acidosis

**DOI:** 10.1002/ccr3.2623

**Published:** 2020-01-07

**Authors:** Prashanth Rawla, Krishna Chaitanya Thandra, John Sukumar Aluru, Sarah Abdel Mageed, Eman Elsayed Sakr, Ghadeer Gamal Elsayed, Mohamed Zidan, Mostafa Ebraheem Morra

**Affiliations:** ^1^ Department of Internal Medicine SOVAH Heath Martinsville VA USA; ^2^ Department of Pulmonary and Critical Care Medicine Sentara Virginia Beach General Hospital Virginia Beach VA USA; ^3^ Senior Research Associate Beth Israel Deaconess Medical Center Harvard Medical School Boston MA USA; ^4^ Department of Internal Medicine Faculty of Medicine Tanta University Tanta Egypt; ^5^ Faculty of Medicine Menoufia University Menoufia Egypt; ^6^ Faculty of Medicine Benha University Benha Egypt; ^7^ Elraml Paediatric Hospital Alexandria Egypt; ^8^ Faculty of Medicine Al‐Azhar University Cairo Egypt

**Keywords:** case report, renal tubular acidosis, systematic review, systemic lupus erythematosus

## Abstract

Immune profile assessment—particularly for SLE—and subsequent specific therapy are beneficial in patients with persisting unexplained hyperkalemic or hypokalemic paralysis, especially in case of isolated RTA.

## Graphical abstract

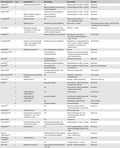

## 1 INTRODUCTION

Although the glomerular involvement in systemic lupus erythematosus (SLE) is widely described in the literature, renal tubular acidosis (RTA) has been rarely reported. We aimed to report a case of RTA in the setting of SLE as well as systematically review all the previously reported cases.

Systemic lupus erythematosus (SLE) is a systemic autoimmune disease that predominantly affects females with an overall 8:1 female‐to‐male ratio; this ratio varies with age to be 7:1 and 15:1 in the elderly and adults, respectively.^1^, ^2^ SLE is a multiorgan disease with a characteristic renal involvement. While glomerular involvement has been widely reported, interstitial involvement (eg, in the form of renal tubular acidosis (RTA)) has been rarely reported; attributable to potassium imbalance in almost all cases. Interstitial diseases used to manifest either prior to or ensuing the diagnosis of SLE. Distal RTA (type 1) together with the inability to concentrate urine, hyporeninemic hypoaldosteronism, and reduced secretion of urinary acid have been observed.^3^, ^4^, ^5^ Accordingly, the diagnosis of SLEis challenging since its criteria may not appear simultaneously. Moreover, RTA has a multitude of differential diagnoses ranging from autoimmune diseases (Sjogren syndrome and rheumatoid arthritis) 6 to other nonautoimmune etiologies like hypercalciuria and drug associations with ifosfamide, amphotericin B, and lithium carbonate.^7^ RTA is characterized by serum potassium imbalance in the setting of normal serum anion gap metabolic acidosis and positive urinary anion gap.^8^ When hypokalemia is the case, the condition may be complicated by weakness of the respiratory muscles up to respiratory arrest; the presentation of RTA is commonly misdiagnosed as hypokalemic periodic paralysis.^9^ Nonetheless, the persistence of hypokalemia in line with the negative family history of hypokalemic periodic paralysis favors RTA diagnosis.^7^

The reason why practices misdiagnose the autoimmune disorders presenting as RTA is that these cases may initially present as hypokalemic paralysis. Of note, the RTA is diagnosed via a combination of hyperchloremic metabolic acidosis and abnormally alkaline urine (PH > 5.5).

Herein, this article systemically depicts the previously reported RTA cases in the setting of SLE together with presenting a new similar case. Although the primary Sjögren's syndrome had been proved to be a common cause for RTA, we would investigate such relation in SLE patients.^10^

In August 2018, an EMBASE, Web of Science, PubMed, and Scopus computerized systematic search was conducted encompassing the terms “systemic lupus erythematosus” and “tubular acidosis OR renal tubular acidosis.” All human studies with relevant data on the association between SLE and RTA were included with no restriction on study design, age, or publication year. Two independent authors screened the yielded articles for inclusion/exclusion. Supplementing the electronic search, the reference lists of the relevant studies were surveyed for further relevance.

## 2 CASE REPORT

In November 2017, an 18‐year‐old female patient presented to the neuropsychiatry department with a week history of progressive lower limb weakness. The patient reported a history of large joints (knee and elbow) arthralgia, for which she received occasional analgesics. There was no malar rash or oral ulcers by examination. She recalled no family history of a similar complaint and had a negative history of illicit drug use or alcohol consumption. Also, she reported a past history of splenectomy as a therapeutic measure for immune thrombocytopenia. The patient had vital signs within normal range. Laboratory evaluation revealed severe hypokalemia (1.5 mmol/L). Electrolyte assessment (serum Na, mg, ionized ca) and thyroid function tests (TSH = 1.18; FT4 = 2.1; FT3 = 1.34) were within normal. She had no history of vomiting or diarrhea. Moreover, electromyography (EMG) demystified mild acute inflammatory demyelinating polyradiculoneuropathy (AIDP). Eventually, the patient was diagnosed with hypokalemic periodic paralysis. Accordingly, she received potassium chloride (100 meq; IV infusion) and was eventually discharged after improvement.

Four months later, the patient was readmitted with a similar attack. Again, the blood workup revealed metabolic acidosis with a potassium level of 2.28 mEq/L and a normal serum anion gap (11 mEq/L). Causes of hypochloremic acidosis like severe diarrhea were excluded. Urine analysis showed alkaline urine (pH 7.5). The patient condition ameliorated after receiving intravenous sodium bicarbonate and potassium infusion. The diagnosis of hypokalemia secondary to distal RTA was considered; then, she was discharged after being scheduled for follow‐up in the general internal medicine clinic. In our patient, we did not perform urinary anion gap calculation since the patient was diagnosed with RTA based on normal serum anion gap, metabolic acidosis, exclusion of vomiting and diarrhea, high urinary potassium. Correction of metabolic acidosis was conducted by NaHco3 which confirmed that it is distal RTA not proximal one. Ultrasound revealed no nephrolithiasis in our patient.

Throughout her follow‐up, the patient was persistently hypokalemic (K level 2‐3 mEq/L) with a mild metabolic acidosis (blood pH = 7.17, HCO_3_ **^‐^**=10.5 mmol/L) and urinary potassium level of 25.8 mEq/L/day. Serum aldosterone and diurnal variation of cortisol were normal. Complete blood count showed persistent leucopenia (TLC 2800‐3500/cumm). Serum urea and creatinine were within normal. The 24‐hour urinary protein was 1540 mg. The autoimmune workup revealed positive both Anti‐double stranded DNA antibody (anti‐dsDNA) (225 IU/mL) and antinuclear antibody (ANA) titer (1/300). Complement proteins C3 and C4 were markedly consumed: 55 mg/dL and 8 mg/dL, respectively. Renal biopsy revealed lupus nephritis, the mesangioproliferative type. The persistent hypokalemic weakness improved dramatically on administering a dose of 40 mg prednisolone daily. Also, the serum potassium level and acidosis normalized with administering sodium bicarbonate and potassium. This emphasized the diagnosis of secondary distal RTA due to systemic lupus erythematosus.

**Figure 1:**
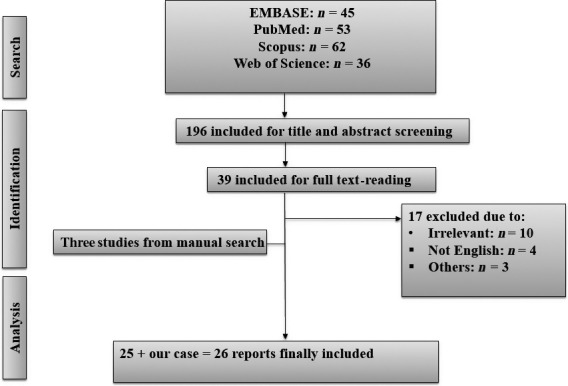
Flow diagram of studies' selection and screening

A search of databases mentioned above revealed a total of 196 relevant articles. Of which, only 26 case reports fit our selection criteria. So, a total of 26 cases were included, including the presented case (Figure 1).

Data from 26 case reports entailing 33 patients were extracted and analyzed. The mean age of the population in these reports was 28.7 years. The overall number of females was 30 (90.9%), and they presented with a multitude of manifestations. Twenty‐six cases were eventually diagnosed with type‐one/distal RTA whereas only six patients were diagnosed with type‐four RTA; our case was type 1 as well. The symptoms at presentation varied greatly between isolated neurologic weakness of the lower limbs, as evident in our case, progressive weakness of both upper and lower limbs, generalized flaccid paralysis, or generalized weakness with massive weight loss. Also, the onset of diagnosis of SLE and RTA in relation to each other had a great variation: SLE has been diagnosed in 13 cases at a point varying from one week to 10 years before diagnosing RTA. Whereas eight cases were diagnosed with SLE at points ranging from one month to six years after diagnosing RTA. Simultaneous diagnosis of SLE and RTA was evident in 11 patients. Of note, our patient was diagnosed with SLE during the RTA follow‐up (Table 1).

**Table 1:** Baseline characters of included reports

First Author/Year	Sample	Age	Sex	Presenting symptoms at admission	Sequence of diagnosis	RTA subtype
Akin/2014^14^	1	20	F	Malaise and edema in the lower extremities and face, polyarthralgia of wrist, hand, feet. She had multiple lymphadenopathies	Both were diagnosed at the same admission	Four
Bagga/1993^4^	1	10	F	Two episodes of generalized tonic‐clonic seizures and painful periarticular swelling of right elbow, left knee, significant cervical, and axillary lymphadenopathy	SLE diagnosed 3 y after RTA	One
Bastani 1997^15^	2	31	F	Nephrotic syndrome and hyperchloremic metabolic acidosis	SLE diagnosed 10 y before RTA	One
		21	F	Elevation in serum creatinine and significant proteinuria	SLE diagnosed 5 y before RTA	One
Caruana/1985^6^	2	25	F	Abdominal pain and numbness and tingling of face and hands	SLE diagnosed 7 y before RTA	One
		48	F	Hypertension and proteinuria	Both were diagnosed at the same admission	One
Chou/2008^16^	1	24	F	Recurrent episodic muscle weakness that progressed to paralysis involving both lower extremities	SLE diagnosed 6 mo after RTA	One
Deng/2016^17^	1	42	F	For management of hyperthyroidism	Both were diagnosed at the same admission	One
Dreyling/1990^18^	1	17	M	Evaluation of renal failure with proteinuria	SLE diagnosed 6 mo before RTA	Four
Fang/2000^19^	1	21	F	Progressive weakness of lower legs	Both were diagnosed at the same admission	One
Fortenberry/1991^20^	1	14	F	Nausea, vomiting, anorexia, malaise, and weight loss	SLE diagnosed 7 mo after RTA	One
Fye/1976^21^	1	57	F	Anorexia, malaise, fever, night sweats, polyarthralgia, pedal edema, dull substernal chest pain, weight loss.	Both were diagnosed at the same admission	One
Gera/2011^22^	1	43	F	Pain in multiple joints	SLE diagnosed 4 y after RTA	One
Gur/1987^23^	1	25	F	Pneumonia and measles	Both were diagnosed at the same admission	One
Hataya/1999^24^	1	12	F	Fever for 3 weeks and diarrhea for few days	Both were diagnosed at the same admission	One
Ranjeet Kaur/2014^25^	1	22	F	Progressive weakness of both upper and lower limbs	Both were diagnosed at the same admission	One
Koul/2003^26^	1	18	F	progressive weakness of all four limbs	SLE diagnosed 3 y after RTA	One
Li/2015^27^	6	25	F	NR	SLE diagnosed 7 y before RTA	One
		29	F	NR	SLE diagnosed 8 y before RTA	One
		26	F	NR	SLE diagnosed 1 year before RTA	One
		22	F	NR	SLE diagnosed 1 year before RTA	One
		31	F	NR	SLE diagnosed 3 y before RTA	Four
		26	M	NR	SLE diagnosed 1 year before RTA	Four
Lim/1987^28^	1	43	F	Intermittent epigastric pain	SLE diagnosed before RTA (duration not declared)	One
Nandi/2016^29^	1	9	F	Failure to thrive for the last 4 y	SLE diagnosed 1 month after RTA	One
Pahadiya/2018^30^	1	65	F	Gradually progressive weakness of both upper and lower limb for 7 days	Both were diagnosed at the same admission	One
Parrey/2018^31^	1	30	F	18‐month history of polyuria and polydipsia	Both were diagnosed at the same admission	One
Porteous/2011^32^	1	44	F	Generalized weakness, weight loss of 40 pounds	Both were diagnosed at the same admission	Four
Prasad/2014^33^	1	30	F	Gradually increasing weakness of both upper and lower limbs	SLE diagnosed 6 mo after RTA	One
Sanchez‐Marcos/2015^34^	1	24	M	Polyarthritis, weakness, myalgia, occasional fever	SLE diagnosed one week before RTA	Four
Ter Meulen CG/2002^35^	1	31	F	(lupus admission) high fever, arthralgia of both ankle, butterfly shaped rash on the face	SLE diagnosed 6 y after RTA	One
Mejía/2017^36^	1	45	F	A three‐hour condition of tachypnea and flaccid quadriparesis	SLE diagnosed 7 y before RTA	One
Our report	1	18	F	Lower limb weakness	SLE diagnosed during follow‐up of RTA	One

Abbreviations: F, female; M, male; NR, not reported; RTA, renal tubular acidosis; SLE, systemic lupus erythematosus.

Most articles have reported PH‐related and potassium values. Serum PH was within normal range (7.35‐7.45) in three cases; meanwhile, 20 cases were acidotic (<7.35), having 10 unreported serum pH values. Also, serum Hco3^‐^ has fallen within the normal range (22‐28 mEq/L) in one patient, and it was subnormal (<22 mEq/L) in 23 cases, having extra nine unreported patient values. Regarding serum potassium level, aside from four unreported values, three cases had a normal range (3.5‐5 mEq/L), 17 cases were hypokalemic (<3.5 mEq/L), and nine were hyperkalemic (<5 mEq/L). Scrutinizing the urinary PH, excluding 12 unreported values, 11 cases had normal urinary pH (6‐7.4), three cases had acidotic urine (>6), and seven patients had alkalotic urine (>7.4).

Histopathological exams revealed membranous glomerulonephritis in five patients and diffuse proliferative glomerulonephritis in other five patients. However, the result of kidney biopsy was not reported in 14 patients. Fifteen patients reported no complications, while hypokalemic paralysis and nephrocalcinosis detected in four and eight cases, respectively. Moreover, renal failure was noticed in four patients. Most of the patients received immunosuppressive drugs, steroids, and/or electrolytes. Only one patient reported death complication while most of the patients (n = 24) improved or at least showed remission of symptoms then were discharged. The outcome was not clearly declared in seven cases (Table 2).

**Table 2:** Renal lesions, complications, intervention, and outcomes among included reports

First Author/Year	Case	Complications	Renal biopsy	Intervention	Outcome
Akin/2014^14^	1	Minimal pericardial effusion	NA	Immunosuppressive drug + steroids	Remission
Bagga/1993^4^	1		Diffuse proliferative and sclerosing glomerulonephritis	Immunosuppressive drug + steroids	Remission
Bastani/1997^15^	1		Membranous glomerulonephritis	Immunosuppressive drug + steroids	Remission
	2	Mild‐to‐moderate congestive HF, renal failure	Diffuse proliferative glomerulonephritis	Immunosuppressive drug + steroids	Remission
Caruana/1985^6^	1	Nephrocalcinosis	NA	Sodium and potassium citrate + magnesium oxide	Remission
	2	Nephrocalcinosis	Membranous glomerulonephritis	Furosemide + steroids	The proteinuria did not improve, but the serum bicarbonate rose to 26 mEq/L
Chou/2008^16^	1	Hypokalemic paralysis, first‐degree AV block, and prominent u waves	Tubulointerstitial nephritis with lymphocytes and plasma cell infiltration and tubular atrophy	Steroids + k citrate	Remission
Deng/2016^17^	1		Diffuse proliferative lupus nephritis	Oral potassium with radioactive iodine. Immunosuppressive drug + steroids.	Not declared
Dreyling/1990^18^	1	Seizures, severe arterial hypertension and congestive heart failure, renal failure	Membranous glomerulonephritis	Hemodialysis + immunosuppressive drug + steroids, b‐blocker, diuretic, ace inhibitor, vasodilator, and fludrocortisone	Not declared
Fang/2000^19^	1	Hypokalemic paralysis	Focal mesangial hypercellularity	Steroids	Remission
Fortenberry/1991^20^	1		Focal proliferative glomerulonephritis	Albumin, isotonic saline,sodium bicarbonate, potassium, bicarbonate, and k supplementation	Remission
Fye/1976^21^	1		Focal proliferative glomerulonephritis	Antibiotics and steroids	improved
Gera/2011^22^	1		Membranous glomerulonephritis	Immunosuppressive drug + steroids	Remission
Gur/1987^23^	1		Focal proliferative glomerulonephritis	Fluids, bicarbonate, hydrocortisone, and antibiotics	Improved and discharged
Hataya/1999^24^	1		Mesangial hypercellularity with mesangial deposits	Prednisone 2mg/Kg Daily	Not declared
Ranjeet Kaur/2014^25^	1	Nephrocalcinosis,hypokalemic paralysis	NA	Antibiotics + potassium supplementation	Not declared
Koul/2003^26^	1	Nephrocalcinosis	NA	Steroids + sodium bicarbonate	Remission, follow‐up
Li/2015^27^	1		Interstitial nephritis	corticosteroids, sodium bicarbonate, and potassium chloride	Remission
	2		NA	corticosteroids, sodium bicarbonate, and potassium chloride	Remission
	3	Nephrocalcinosis, nephrolithiasis	NA	Corticosteroids	Not declared
	4	Nephrocalcinosis, nephrolithiasis	Interstitial nephritis	corticosteroids, sodium bicarbonate, and potassium chloride	Remission
	5	Renal failure	NA	Hemodialysis, hydrocortisone	Died eventually.
	6		Interstitial nephritis	Corticosteroid only	Not declared
Lim/1987^28^	1		NA	Resonium a, shohl's solution (nahco3 and citrate) + furosemide	Remission
Nandi/2016^29^	1	Nephrocalcinosis	NA	Sodium bicarbonate	
Pahadiya/2018^30^	1	Central pontine myelinolysis, flattening of t waves and prominent u waves.	NA	Sodium bicarbonate tab, syrup potassium chloride, and oral prednisolone	Remission
Parrey/2018^31^	1		NA	Vasopressin	Not declared
Porteous/2011^32^	1	Pericardial effusion, pleural effusion, renal failure	Diffuse global proliferative and membranous glomerulonephritis	Immunosuppressive drug + steroids	Remission
Prasad/2014^33^	1	Nephrolithiasis	NA	K, hco3 supplementation + steroids hydroxychloroquine 200 mg twice a day	Remission
Sanchez‐Marcos/2015^34^	1		Diffuse proliferative lupus nephritis and 25% interstitial nephritis	Immunosuppressive drug + steroids+acei	Remission
Ter Meulen CG/2002^35^	1	Nephrocalcinosis	NA	Steroids	Remission
Mejía/2017^36^	1		NA	K supplementation + steroids	Remission
Our report	1	Hypokalemic paralysis	Lupus nephritis mesangioproliferative type	Steroids + sodium bicarbonate	Remission

## 3 DISCUSSION

The literature has reported few cases of RTA in the context of SLE, especially the distal type (type 1) with fewer reports on the association with proximal RTA (type 2). The diversity of systematic manifestations of SLE, and the challenge in recognizing and approaching such manifestations prior to establishing SLE's diagnosis necessitate a thorough assessment to provoke clinical suspension in similar settings. In our review, we reported a rare case of type 1 RTA presenting with lower limb weakness prior to diagnosing SLE. While renal glomerular injury is the major finding in lupus nephritis, renal tubular injury has been rarely reported.^11^

Each type of RTA is a separate entity with unique characteristics and therapeutic measures. Type 1 (distal RTA) is mostly associated with hypokalemia while (type 4) is associated with hyperkalemia.^12^ Distal RTA is mostly a genetic disorder that presents in childhood; however, it occurs in adolescence secondary to other disorders like auto‐immune dysfunctions.^13^ RTA has a wide range of presentations either neurologic (isolated lower limb weakness, generalized weakness, quadriparesis, etc), nonspecific GI tract symptoms (epigastric pain, weight loss, joint pain, etc), or even the symptoms of the primary disorder (eg, discoid rash or proteinuria in case of SLE). Accordingly, the diagnosis of secondary RTA and its related primary etiology is troublesome. While RTA is mainly a biochemical diagnosis, the treatment is not restricted to correcting/balancing the biochemical values/electrolyte level, but rather it includes treatment of the cause, and is dependent on recognizing the type and pathology of the acidosis. For example, practices should consider early steroid use for SLE and most autoimmune diseases to curtail recurrences. Therefore, it is important to thoroughly investigate any renal symptoms or general symptoms that depict renal injury to avoid any delays in treatment or any misdiagnoses. In addition, it is vital to rule out any plausible causes of renal tubular acidosis prior to the initiation of any therapy.

Of the 36 reported SLE‐RTA associations, our case, as well as 27 previous cases, had type 1 RTA. That is confirmed by the dramatic reversal of the serum potassium and acid levels to normal values with sodium bicarbonate and potassium administration. Similar to eight other reported cases, our patient was diagnosed with SLE following RTA diagnosis, which made it harder to initially consider SLE as the primary etiology. Based on our case, the diagnosis of SLE in a previously diagnosed RTA patient established on the ground of significant proteinuria that led to immunology workup (anti‐double stranded DNA antibody, rheumatoid factor (RF), antinuclear antibody (ANA) titer, and complement proteins C3 and C4 levels).

## 4 CONCLUSION

As the immune profile confers a considerable prebiopsy evaluation of the primary disorders, clinicians are recommended to consider assessing it in patients with persisting unexplained hyperkalemia or hypokalemic paralysis; moreover, we recommend an emphasis on autoimmune diseases, especially SLE, in patients with isolated RTA. Further studies are needed to investigate the strength of correlation and to better understand the underlying etiology of such association.

## CONFLICT OF INTEREST

The authors declare no conflict of interest.

## AUTHOR CONTRIBUTIONS

PR, SA, EES, GGE, MEZ, MEM: conceived and designed the study. PR, KCT, JSA, SA, EES, GGE, MEZ, MEM: acquired, analyzed, and interpreted the data. PR, KCT, JSA, SA, EES, GGE, MEZ, MEM: drafted the manuscript. PR, KCT, JSA, SA, EES, GGE, MEZ, MEM: final approval of the manuscript.

## HUMAN AND ANIMAL RIGHTS

This article does not contain any studies with human participants or animals performed by any of the authors.

## INFORMED CONSENT

Informed consent was obtained from all individual participants included in the study.
